# Analysis of global transcriptional responses of chicken following primary and secondary *Eimeria acervulina* infections

**DOI:** 10.1186/1753-6561-5-S4-S12

**Published:** 2011-06-03

**Authors:** Chul-Hong Kim, Hyun S Lillehoj, Yeong-Ho Hong, Calvin L Keeler, Erik P Lillehoj

**Affiliations:** 1 Animal Parasitic Diseases Laboratory, Animal and Natural Resources Institute, Agricultural Research Service, USDA, Beltsville, MD 20705, USA; 2 Department of Animal and Food Sciences, College of Agriculture and Natural Resources, University of Delaware, Newark, DE 19716, USA; 3 Department of Pediatrics, University of Maryland School of Medicine, Baltimore, MD 20201, USA

## Abstract

**Background:**

Characterization of host transcriptional responses during coccidia infections can provide new clues for the development of alternative disease control strategies against these complex protozoan pathogens.

**Methods:**

In the current study, we compared chicken duodenal transcriptome profiles following primary and secondary infections with *Eimeria acervulina* using a 9.6K avian intestinal intraepithelial lymphocyte cDNA microarray (AVIELA).

**Results:**

Gene Ontology analysis showed that primary infection significantly modulated the levels of mRNAs for genes involved in the metabolism of lipids and carbohydrates as well as those for innate immune-related genes. By contrast, secondary infection increased the levels of transcripts encoded by genes related to humoral immunity and reduced the levels of transcripts for the innate immune-related genes. The observed modulation in transcript levels for gene related to energy metabolism and immunity occurred concurrent with the clinical signs of coccidiosis.

**Conclusions:**

Our results suggest that altered expression of a specific set of host genes induced by *Eimeria* infection may be responsible, in part, for the observed reduction in body weight gain and inflammatory gut damage that characterizes avian coccidiosis.

## Background

The apicomplexan *E.acervulina* specifically infects the duodenum resulting in diarrhea, poor feed conversion, and reduced body weight gain, thereby incurring large economic losses to the poultry industry [1]. Traditional disease control methods have relied on chemoprophylaxis with anti-coccidia drugs or immunization with live/attenuated parasite vaccines [2]. However, novel strategies are sought due to increasing governmental restrictions on the commercial use of coccidiostats, the emergence of drug resistant parasites, and the high costs of new drug/vaccine development. Recent high throughput DNA microarray technology on a whole-genome or tissue-specific basis enables the investigation of complex transcriptional patterns, providing new insights to analyze intricate biological systems, such as host-parasite interactions during coccidiosis [3]. Recently, we constructed a 9.6K intestinal IEL cDNA microarray (AVIELA) which was utilized to show that immune-related genes such as apoptosis as well as the JAK/STAT and MAPK signaling pathways were up- or down-regulated in the jejunum during *E. maxima* infection [4]. The current study was undertaken using the improved second-generation AVIELA to study local immune responses in chickens infected with a duodenum-infecting coccidia species, *E. acervulina*, since its host immune response is less known compared to other *Eimeria* parasites.

## Methods

### Animals, parasites, and experimental infections

Fertilized eggs of White Leghorn chickens (Charles River SPAFAS Laboratories) were hatched at the Animal and Natural Resources Institute (Beltsville, MD). *E*. *acervulina* oocysts were cleaned by flotation on 5.25% sodium hypochlorite and washed 3 times with sterile PBS [5]. Chickens were orally inoculated with 1.0 X 10^4^ sporulated oocysts of *E. acervulina* at 3 weeks of age. Secondary infection with 2.0 X 10^4^ oocysts was given at day 21 post primary infection (i.e. 6 weeks of age). Non-infected 3 week-old and 6 week-old chickens were used as primary and secondary negative controls, respectively. All experiments were approved by the Institutional Animal Care and Use Committee (IACUC) at Animal and Natural Resources Institute.

### Isolation of intestinal mucosal tissues, RNA preparation, microarray hybridization, and data analyses

Construction of the AVIELA microarray and isolation of intestinal mucosal tissues were previously described [4,6]. Intestinal duodenal tissues were removed from 12 chickens/group at daily intervals between days 0 and 8 post-primary or post-secondary infections. The scrapings of mucosal layers from 12 chickens from each group were pooled and total RNA was prepared using TRIzol (Invitrogen) and the RNeasy Mini RNA Purification Kit (Qiagen). The pooled total RNA was used for microarray hybridization and quantitative RT-PCR. DNase-treated total RNA (3.0 μg) from 2 consecutive days were pooled (days 1-2, 3-4, 5-6, and 7-8) and used for synthesis of aminoallyl-labeled RNA (aRNA) using the Amino Allyl Message Amp II aRNA Amplification Kit according to the manufacturer’s protocol (Ambion, Austin, TX). Two 15 μg aliquots of each aRNA were fluorescently labeled with Alexa Fluor 555 or Alexa Fluor 647 (Invitrogen). For hybridization, a circular loop design was employed with technical replication without dye-swap (day 0 vs. days 1-2, days 1-2 vs. 3-4, days 3-4 vs. 5-6, and days 5-6 vs. 7-8) for primary and secondary infections [7,8]. The microarray data and bioinformatics analyses were performed as previously described [4]. The MIDAS 2.19 of the TM4 package (http://www.tigr.org) was used to qualify and normalize the array data. The poor-quality channel tolerance policy was stringent and the signal-to-noise threshold was 2.0. Two-step normalization, total intensity and global LOWESS (locally-weighted regression and smoothing scatter plots) methods were applied followed by standard deviation (SD) regularization between blocks and slides. The qualified and normalized array data were transferred to GeneSpring GX 7.3 (Silicon Genetics, Redwood, CA) for fold change and statistical analyses. The elements that were modulated ≥ 2.0-fold during primary infection or 1.5-fold during secondary infection were filtered using the Volcano plot to assess statistically significance (*p* < 0.05). The microarray data and additional information were registered at the NCBI GenBank Gene Expression Omnibus (GEO) repository, series accession number GSE16230.

### Quantitative RT-PCR

To confirm gene expression changes observed by microarray analysis, qRT-PCR was performed as previously described [9] with *B2M*, *CD3D*, *FBP1*, *IGJ*, *IL16*, and *SOD1* using Mx3000P system and Brilliant SYBR Green QRT-PCR master mix (Stratagene, La Jolla, CA). Standard curves were generated using 2-fold diluted standard RNA and the levels of individual transcripts were normalized to those of GAPDH analyzed by the Q-gene program [10]. To normalize RNA levels between samples within individual experiments, the mean threshold cycle value (C_t_) for the target gene and GAPDH products were calculated by pooling values from all samples in that experiment. Statistical test of qRT-PCR was performed using Microsoft Excel 2003 and student T-test was used to test for the group differences.

## Results

### Status of modulated genes during primary and secondary *E. acervulina* infections

**Figure 1:**
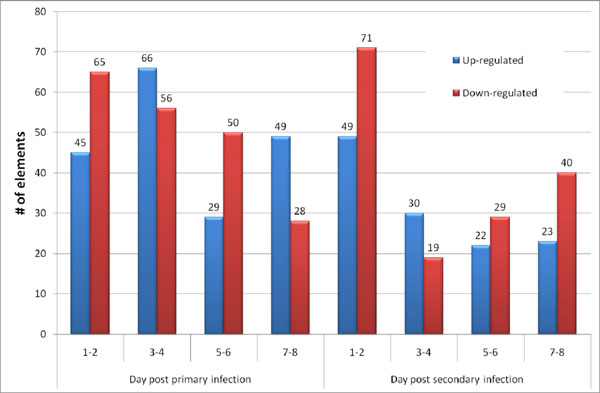
Summary of significantly up- or down-regulated elements (p < 0.05) during primary or secondary *E. acervulina* infections.

Because the variances in fold changes of elements were large during primary infection, the cut-off value for statistical significance was assigned as ≥ 2.0-fold for increased expression and ≤ 0.5-fold for decreased expression, respectively. In the case of secondary infection, variance was relatively lower and cut-off values were ≥1.5-fold and ≤ 0.66-fold for up- and down-regulated genes, respectively. The total numbers of up- or down-regulated transcripts during primary infection were larger than those altered during secondary infection, 189 vs. 124 up-regulated transcripts during primary vs. secondary infection and 199 vs. 159 down-regulated transcripts during primary vs. secondary infection (Fig 1)

### Gene Ontology analysis of up- and down-regulated genes during *E. acervulina* infections

**Figure 2:**
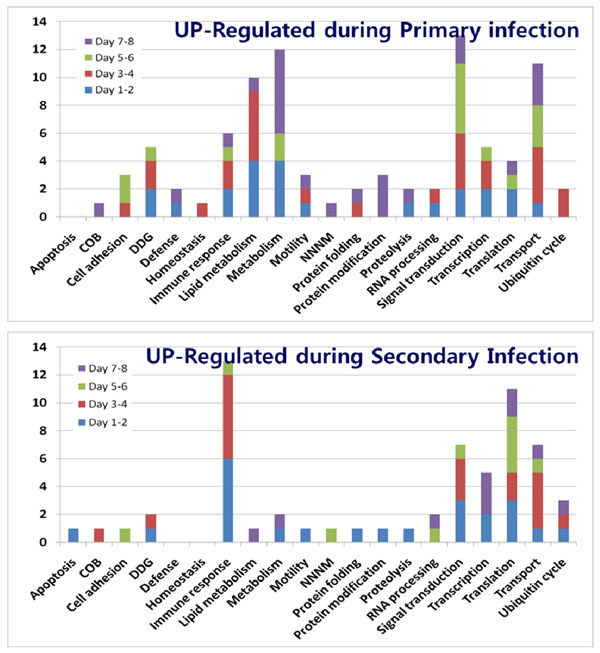
**Biological processes of Gene Ontology of up-regulated genes during primary or secondary *E. acervulina* infections.** COB, cell organization and biogenesis, DDG, development, differentiation, and growth; NNNM, nucleoside, nucleotide and nucleic acid metabolism.

**Figure 3:**
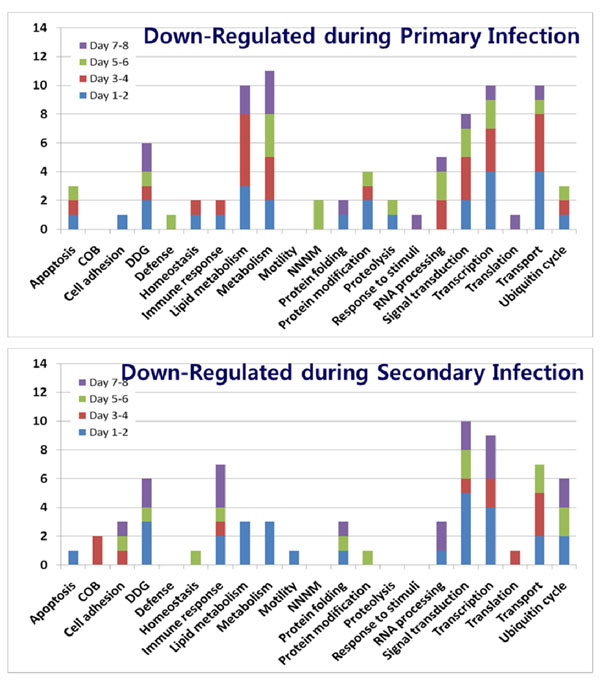
**Biological processes of Gene Ontology of down-regulated genes during primary or secondary *E. acervulina* infections**. COB, cell organization and biogenesis, DDG, development, differentiation, and growth; NNNM, nucleoside, nucleotide and nucleic acid metabolism.

Functional analysis of gene profiles provides a global view of host-parasite interactions and pathogenic mechanisms during avian coccidiosis. As shown in Figures 2 and 3, GO analysis showed that the greatest number of altered levels of transcripts occurred for genes involved in lipid metabolism, general metabolism, immune response, signal transduction, transcription, translation, and transport. However, when comparing primary vs. secondary infections, it was readily apparent that while transcripts of genes for metabolism were highly altered during primary infection, those for immunity were modulated to a greater extent during secondary infection. Transcripts for genes related to signal transduction, transcription, translation, and transport generally appeared to be altered to equivalent extents during primary and secondary infection. To classify significantly changed biological processes during *E. acervulina* infections, the GO processes identified in Figures 2 and 3 were further analyzed using EASE program according to biological processes with terms at level 4 and significance analyses with a minimum of 2 genes at FDR < 0.1. EASE provides statistical methods for discovering enriched biological themes within gene lists [11]. During primary infection, genes involved in carbohydrate metabolism (*p* < 0.001), cell-cell adhesion (*p* < 0.05), lipid metabolism (*p* < 0.001), response to oxidative stress (*p* < 0.05), and immune response/defense (*p* < 0.001) were identified as the most affected, whereas during secondary infection only immune response/defense-related genes were identified (*p* < 0.001) (data not shown).

### Validation of AVIELA analysis by qRT-PCR

Expression patterns observed by microarray analysis were validated by qRT-PCR with 6 transcripts whose levels were significantly modulated during primary or secondary *E. acervulina* infections (*B2M*, *CD3D*, *FBP1*, *IGJ*, *IL16*, *SOD1*). According to the microarray, the levels of these 6 mRNAs were significantly altered at 10 time points post-primary or post-secondary infection as assessed using the AVIELA. Similarly, all transcripts at these 10 times also were significantly changed in the same manner (up or down) when analyzed by qRT-PCR (Table 1).

**Table 1:** Comparison of microarray with QRT-PCR data

		QRT-PCR	Microarray
		
Gene symbol	Day^a^	Fold change with pvalue	Fold change
*B2M*	5-6 DPI	1.5 (0.01)	2.1
	1-2 DSI	1.4 (0.01)	1.8
*CD3D*	1-2 DPI	1.7 (0.01)	2
*FBP1*	1-2 DPI	1.6 (0.01)	2.1
	5-6 DPI	0.5 (0.01)	0.36
	7-8 DPI	3.8 (0.001)	2.3-3.4
*IGJ*	1-2 DPI	0.35 (0.01)	0.48
	5-6 DSI	0.31 (0.001)	0.36-0.39
*IL16*	7-8 DSI	0.49 (0.01)	0.58
*SOD1*	3-4 DPI	1.7 ( 0.001)	2.7

^a^DPI, days post-primary infection; DSI, days post-secondary infection

## Conclusions

This study describes the first report of comparing primary and secondary local global transcriptional responses elicited in the duodenal mucosa following *E. acervulina* infection. The observed gene expression showed overall responses from various different cell types in duodenum intestine, not from specific cell types that resulted from trafficking or turning over during infection. However, our findings indicated that primary infection modulated the levels of transcripts for genes involved in and lipid/carbohydrate metabolism as well as innate and cellular immunity while secondary infection was associated with changes in transcripts for genes of humoral immunity. These results illustrate the utility of the AVIELA microarray for elucidating the differential mechanisms employed by chickens in responding to *Eimeria* infection at the transcriptome level and, as such, will provide valuable information for further characterization of host protective immunity to avian enteric pathogens. More importantly, the genes identified in this report may represent novel targets for future genetic modification strategies at the organism level to counteract the effects of coccidiosis.

## Abbreviations

aRNA: aminoallyl-RNA; EASE: Expression Analysis Systematic Explorer; EST: expressed sequence tag; GO: Gene Ontology; HBSS: Hank’s balanced salt solution; IEL: intraepithelial lymphocyte; qRT-PCR: quantitative RT-PCR; FDR: false discovery rate.

## Competing interests

The authors declare that they have no competing interests.

## Authors' contributions

CHK; microarray experiment, statistical and bioinformatic analyses, qRT-PCR, HSL; experiment design, animal experiment, YHH; RNA preparation and qRT-PCR, CLK and EPL; experiment design, revision of experiment data
